# Spaceflight Activates Autophagy Programs and the Proteasome in Mouse Liver

**DOI:** 10.3390/ijms18102062

**Published:** 2017-09-27

**Authors:** Elizabeth A. Blaber, Michael J. Pecaut, Karen R. Jonscher

**Affiliations:** 1 Universities Space Research Association, Mountain View, CA 94040, USA; e.blaber@nasa.gov; 2 NASA Ames Research Center, Moffett Field, CA 94035, USA; 3 Department of Basic Sciences, Division of Radiation Research, Loma Linda University School of Medicine, Loma Linda, CA 92350, USA; mpecaut@llu.edu; 4 Department of Anesthesiology, University of Colorado Anschutz Medical Campus, Aurora, CO 80045, USA

**Keywords:** spaceflight, autophagy, proteasome, metabolomics, tRNA biosynthesis, senescence

## Abstract

Increased oxidative stress is an unavoidable consequence of exposure to the space environment. Our previous studies showed that mice exposed to space for 13.5 days had decreased glutathione levels, suggesting impairments in oxidative defense. Here we performed unbiased, unsupervised and integrated multi-‘omic analyses of metabolomic and transcriptomic datasets from mice flown aboard the Space Shuttle Atlantis. Enrichment analyses of metabolite and gene sets showed significant changes in osmolyte concentrations and pathways related to glycerophospholipid and sphingolipid metabolism, likely consequences of relative dehydration of the spaceflight mice. However, we also found increased enrichment of aminoacyl-tRNA biosynthesis and purine metabolic pathways, concomitant with enrichment of genes associated with autophagy and the ubiquitin-proteasome. When taken together with a downregulation in nuclear factor (erythroid-derived 2)-like 2-mediated signaling, our analyses suggest that decreased hepatic oxidative defense may lead to aberrant tRNA post-translational processing, induction of degradation programs and senescence-associated mitochondrial dysfunction in response to the spaceflight environment.

## 1. Introduction

Long-duration spaceflight is associated with significant risks including prolonged exposure to microgravity, continuous exposure to low-dose/low-dose rate radiation, psychological and environmental stress and contact with potentially dangerous levels of microbial contamination [1,2,3,4,5]. Radiation is known to induce single (SSB) and double strand (DSB) DNA breaks, permanently damaging nuclear and mitochondrial DNA and leading to early apoptosis or necrosis [6,7,8]. At low-dose/low-dose rates, radiation immediately triggers oxidative stress via a spike in the level of reactive oxygen species (ROS). Acute psychological stress increases hepatic lipid peroxidation as well as levels of ROS [9], however chronically stressed animals compensate [9,10]. Therefore, exposure to the space environment, characterized by changes in physiological and psychological stress, as well as exposure to low-dose/low-dose rate radiation, may systemically alter ROS levels in a complex fashion [11,12,13].

Excessive ROS production, without a corresponding upregulation in antioxidant or ROS scavenger pathways, can cause damage to cellular components including DNA, proteins and lipids, inducing pro-inflammatory cytokines and the nuclear factor κ-light-chain-enhancer of activated B cells (NF-κB) pathway [14]. This can lead to cell cycle arrest, activation of senescence or apoptosis and upregulation of inflammatory signaling molecules, causing widespread organelle, cell and tissue damage [15]. Under conditions of chronic exposure to oxidative stress (such as in spaceflight), an imbalance occurs between ROS production and antioxidant quenching resulting in increased cellular and tissue damage [16,17,18]. Mitochondria are particularly vulnerable to damage by excess ROS and we hypothesized that the liver, a mitochondria-rich metabolic organ, may be a target of spaceflight-induced deficits. In support of this hypothesis, astronauts were shown to exhibit a mild diabetogenic phenotype following spaceflight, the severity of which was linked with flight duration [19,20].

We previously demonstrated that mice flown aboard the Space Shuttle Atlantis (Space Transportation System (STS)-135) for 13.5 days exhibited a significantly impaired response to oxidative stress evidenced by decreased hepatic levels of the antioxidant glutathione (GSH, reduced), with concomitant increased ophthalmate, a biomarker for depletion of glutathione, and increased ratio of glutathione disulfide (GSSG):GSH [21]. Other spaceflight-induced changes in hepatic genes linked to oxidative defense have also been observed [13]. Our targeted analyses of metabolomics and transcriptomics datasets obtained from livers of spaceflight mice showed dysregulation of pathways involved in both lipid metabolism and the immune response, with signs of retinoid export and activation of peroxisome proliferator-activated receptor (PPAR) pathways suggestive of nascent hepatic fat accretion and collagen deposition. Proteomics data acquired from mice exposed to space for 30 days exhibited similar patterns when compared with mice re-acclimated to ground conditions [22].

Our previous integrated data analysis utilized a limited set of genes and metabolites that were elevated in abundance in spaceflight mice as compared with ground controls and were tightly correlated with histological evidence of increased hepatic lipid accumulation. The goal of the present study was to perform unbiased, integrated analyses using the entire metabolomics and transcriptomics datasets to determine whether additional insights into the effects of exposure to the space environment on liver metabolism and cellular function could be gleaned. Utilizing several enrichment algorithms, we determined that degradation and senescence programs were altered in spaceflight mice in concert with attenuation of oxidative defense networks.

## 2. Results and Discussion

### 2.1. Short Duration Exposure to the Space Environment Significantly Alters Hepatic Metabolite Profiles

**Figure 1:**
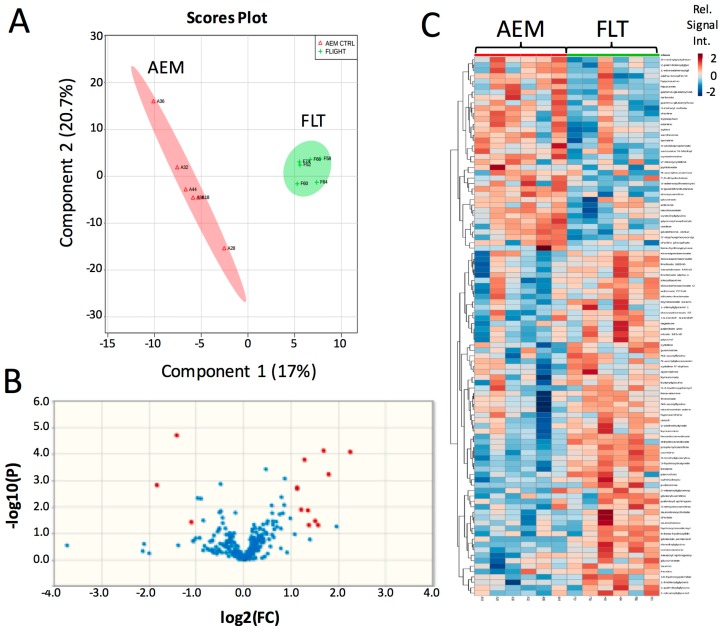
Brief exposure to the space environment results in significant metabolite changes in mouse liver. (**A**) PLS-DA analysis was performed on normalized metabolomics data that was subsequently log-transformed and auto-scaled. The first two components are plotted; (**B**) volcano plot comparing flown in space (FLT) vs. matched ground controls (AEM) considering unequal variance and using a fold change (FC) threshold of 2 and a *p (corr)*-value threshold of 0.05. Data points in red indicate significant named biochemical features. Data points in blue are not significant; (**C**) heat map and hierarchical clustering performed using a Pearson score for the distance measure, with features organized by VIP score from the PLS-DA analysis. Red—AEM, green—FLT; red indicates compounds with high signal abundance and blue those with low signal abundance. Color intensity correlates with relative signal abundance. *n* = 6/group.

Previously, we selected specific genes and metabolites to interrogate from our large-scale-‘omics datasets to address defined hypotheses. Here, we performed unsupervised Partial Least Squares Discriminant Analysis (PLS-DA) using the entire metabolomics dataset to determine whether 13.5 days of exposure to the space environment was sufficient to induce significant changes in metabolism in livers of mice flown in space (FLT) as compared with matched ground controls (AEM). The scores plot of the PLS-DA analysis demonstrates clear separation between the FLT mice (green) and AEM controls (red) (Figure 1A). Volcano plot analysis (Figure 1B) shows that 10 biochemicals increased in FLT mice with a fold change (FC) greater than 2 and a *p (corr)*-value less than 0.05, while only 3 biochemicals decreased in the FLT mice as compared to the AEM controls (Table 1) from a total of 14 upregulated (FC > 2) and 11 downregulated (FC < 0.5) biochemicals (Figure S1A and Table S1). Variable Influence on Projection (VIP) analysis of the top 15 most important features contributing to the clustering is plotted in Figure S1B and the most significant features in the volcano plot also appear within the top 15 VIP features. Hierarchical clustering with organization of features by VIP score (Figure 1C) also shows clear separation of metabolite features between the two groups, with relatively fewer metabolites decreased in abundance in the FLT mice as compared with the AEM controls.

### 2.2. Altered Betaine and Glutathione Metabolism Are Central Defects in Spaceflight Mouse Livers

**Figure 2:**
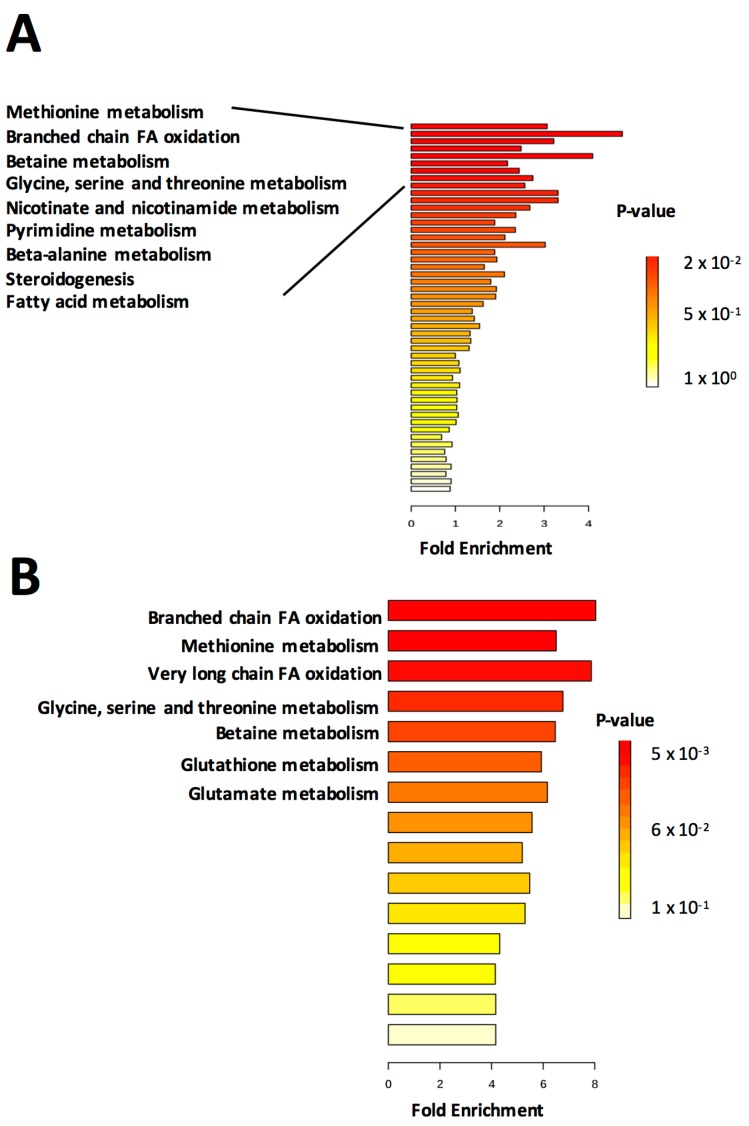
Metabolite set enrichment analysis reveals metabolic pathways enriched in livers of FLT mice. Enrichment analysis was performed in MetaboAnalyst using (**A**) all metabolites or (**B**) only metabolites with significantly changing abundances between groups (*p* < 0.05). *n* = 5/group. Eighty-eight metabolite sets based on “normal metabolic pathways” were used for the analysis. For clarity of presentation, only the most significantly enriched metabolite sets are annotated.

Metabolite set enrichment was performed in MetaboAnalyst using pathway-associated metabolite sets based on “normal metabolic pathways” (Figure 2). Enrichment was performed using either all metabolites (Figure 2A) or a more limited subset consisting only of those significantly changing in abundance (Figure 2B). Significance was determined by two-tailed Student’s *t*-test. A fold-change cutoff was not applied, therefore some of the significant metabolites differ from those identified in the volcano plot. When enrichment analysis was performed using all metabolites, relatively few *p (corr)*-values for enriched metabolite sets reached significance (*p* < 0.05). However, the top several sets (*methionine metabolism*, *branched chain fatty acid oxidation*, *betaine metabolism* and *glycine*, *serine and threonine metabolism*) were retained when the limited subset of metabolites was used for analysis (Figure 2B). Furthermore, enrichment of *glutathione* and *glutamate metabolism*, important components of the response to oxidative stress, rose to significance in the analysis of the limited dataset, suggesting the utility of performing the same analysis multiple times on different subsets of the data.

We next identified specific metabolites that were contributing to the enrichment scores. The *glycine, serine and threonine metabolism* enrichment score was based on decrease of cystathionine and increase of betaine and dimethylglycine. Eight other metabolites contributed to the enrichment, however their abundance changes were not significant between groups. The *glutathione metabolism* enrichment score was based on decreased abundance of glutathione and cysteinylglycine in FLT mice as compared with AEM controls, with additional contributions from four non-significantly changing metabolites. *Fatty acid oxidation* enrichment scores (both *branched chain* and *very long chain*) were based on abundance of propionylcarnitine and carnitine, with coenzyme A and acetyl carnitine contributing to the score as well. It should be noted that MetaboAnalyst does not recognize many lipids, therefore lipid metabolic pathways are likely under-represented. Contributors to enrichment of *methionine metabolism* were similar to those of *betaine metabolism* and consisted primarily of betaine, dimethylglycine and choline, with additional contributions from *S*-adenosylhomocysteine and 5-methyltetrahydrofolic acid, suggesting activation of the *S*-adenosyl methionine (SAM) cycle in the FLT mice. Abundances of these metabolites in FLT mouse livers and AEM controls are summarized in Table S2. For validation, metabolite pathway analysis was performed and similar pathways were found to have a high impact score based on enrichment and topology analysis (Figure S2).

We previously reported a significant increase in abundance of betaine in FLT livers [4,5]. Betaine is metabolized from choline, which decreased in FLT mice [4,5]. Betaine is also a methyl donor and provides the methyl group for metabolism of homocysteine to methionine, generating dimethylglycine as well. Furthermore, the transmethylation cycle provides substrates used for the synthesis of cystathionine and GSH via transsulferation [23]; labile methyl groups are required for these processes as well as support of folate metabolism and synthesis of methylated compounds. Since methionine is lost in the transmethylation pathway, it is possible that choline is supplied to preserve pathway function [24]. However, the observed increase in abundance of betaine with a concomitant decrease in cystathionine and GSH suggests increased choline metabolism in spaceflight is not linked to augmented activation of one carbon metabolic pathways, since abundance levels of relevant metabolites such as *S*-adenosylhomocysteine, serine, sarcosine and glycine are unchanged. Therefore, other mechanisms are likely involved.

**Figure 3:**
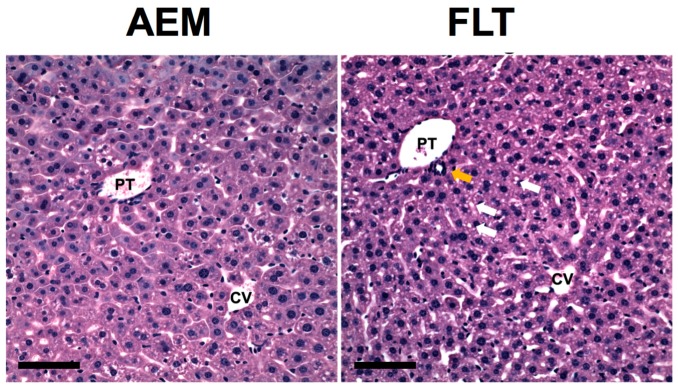
Increased steatosis and infiltration of inflammatory cells in livers from mice exposed to spaceflight. H&E staining was performed on fixed liver sections from *n* = 4–5 mice/group to investigate liver histology. Inspection of the H&E stained sections revealed that the AEM ground control mice had small cytoplasmic lipid droplets predominantly located in zone 2 whereas the FLT mice had an increase in slightly larger droplets distributed in a panlobular pattern. Multiple lipid droplets are indicated using white arrows. Furthermore, FLT mice showed increased accumulation of mononuclear inflammatory cells, particularly near portal ducts (yellow arrow). Representative images are shown from each group. PT = portal triad, CV = central vein. Scale bar = 100 μm.

Dietary choline and methionine induce lipotrophic effects through upregulation of very low-density lipoprotein export and fatty acid oxidation, and choline deficiency has been associated with oxidative stress, inflammation, and steatosis. Shown in Figure 3, histological assessment confirms that spaceflight indeed results in increased presence of inflammatory cells as well as augmented steatosis. Although we previously measured upregulation of mRNA expression levels of PPAR-α, a transcriptional regulator of fatty acid oxidation, it is likely that PPAR activation is mediated through increased retinol abundance instead of elevated choline [5]. Choline may be replenished by recycling from phosphatidylcholine, which may impair integrity of lipid membranes. Metabolomics analysis of choline-containing lysolipids revealed an average 40% decrease in abundance of these lipids in FLT mice as compared to AEM controls, although differences between groups were not significant for individual features (Figure S3). These results suggest that augmented metabolism of betaine, in excess of what is needed for methionine metabolism, may lead to injurious choline deficits in spaceflight mice. Whether this is causally related to decreased glutathione and the ability to respond to oxidative stress remains an open question.

Notably, betaine serves as an organic osmolyte, protecting cells from effects of dehydration. Water intake in FLT mice was decreased by ~20% as compared to AEM controls, although food intake was unchanged (Table 2), therefore a likely cause for the upregulation of betaine is dehydration. We also measured upregulation of taurine, another osmolyte. Abundance of 4-guanidinobutanoate was strikingly decreased (Figure S1A) and was the most important feature contributing to the clustering of groups in the PLS-DA analysis (Figure S1B). Guanidino compounds are metabolized from arginine, and dehydration modifies abundance of these compounds in the kidney [25]. Kidney injury not only results in reduced arginine synthesis but change in levels of guanidino compounds and their metabolism in muscles and liver [26]. The marked decrease in abundance of 4-guanidinobutanoate may therefore be associated with dehydration in the FLT mice. Many of the most striking changes in metabolite and transcript abundances appeared to be related to increased dehydration of the FLT mice as compared to AEM controls; although this is a possible limitation of the study, a recent proteomics study on livers from male mice in the “Bion-M1” study flown in space for 30 days and re-acclimated showed results similar to those from our previously published data [22]. Therefore, either the Bion-M1 mice were also dehydrated, or the enriched pathways that we measured indeed have functional significance, potentially related to a shift in metabolic requirements due to unloading. Taken together, the data suggest that dehydration, coupled with oxidative stress, combine to deplete choline stores, leading to impaired lipid membrane metabolism and contributing to increased steatosis in livers from mice exposed to spaceflight.

### 2.3. Spaceflight Causes Broad Alterations in Transcriptome Profiles in the Liver

**Figure 4:**
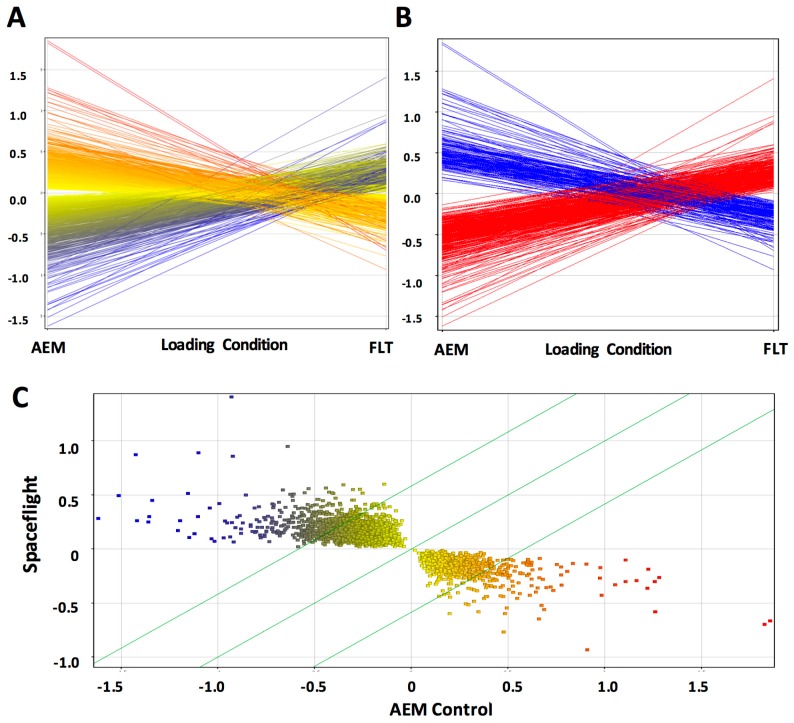
Hepatic gene expression is significantly altered by exposure to the space environment. Profile (**A**) and scatter (**C**) plots of all significantly regulated genes (*p* (*corr*) < 0.05), and (**B**) profile plots of biologically significant genes with differential regulation FC +/− 1.5. Red lines (**B**) and points (**C**) indicate significantly upregulated genes in AEM mice, whilst blue indicate significantly upregulated genes in FLT. Yellow indicates genes that are statistically but not biologically significant.

To understand the role of transcriptional regulation in the observed alterations to metabolites, we performed an unbiased analysis of transcriptome datasets obtained with Affymetrix Genechip 1.0 ST arrays using GeneSpring software. We found significant alterations (*p* (*corr*) < 0.05) in 3005 out of 28,852 genes, or approximately 10% of identifiable probesets (Figure 4A,C). Of these, 601 genes were found to have biological significance; expression levels of 449 genes were upregulated (FC > 1.5) and 152, downregulated (FC < −1.5) (Figure 4B).

**Figure 5:**
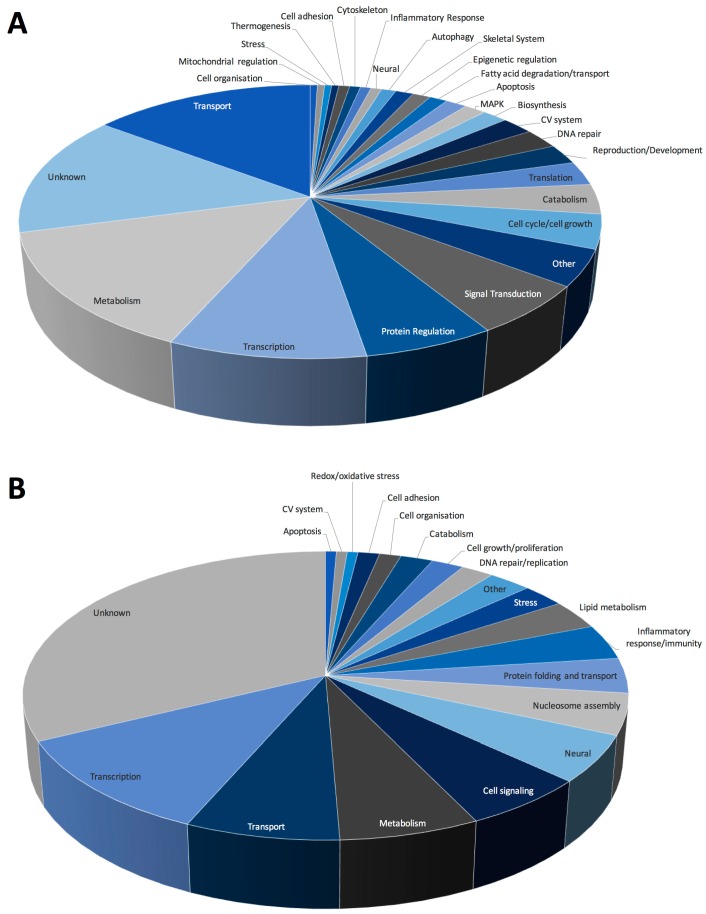
GO biological functions associated with (**A**) up- and (**B**) downregulated datasets. Change in regulation was determined as the ratio of average expression of FLT to AEM values for each transcript, *n* = 6/group.

Analysis of Gene Ontology (GO) biological functions (Figure 5) revealed that most upregulated genes were involved in metabolism or basic cellular processes, including transcription, translation, and DNA repair. Of note, several autophagy-related genes were altered as were genes involved in oxidative stress and regulation of peroxisomes, in particular fatty acid synthesis and degradation (Figure 5A). Our previous studies found significant alterations in both mRNA and metabolites associated with activation of PPAR-mediated pathways, as well as alterations in fatty acid oxidation in response to spaceflight [4,5]. Furthermore, as peroxisomes catalyze redox reactions and are potential regulators of oxidative stress-mediating signaling pathways, it is possible that peroxisomes and mitochondria may cooperate to determine cell fate decisions [27]. Specifically, peroxisomes house several enzymes that can produce or degrade ROS and reactive nitrogen species (RNS) and therefore may act as modulators of oxidative balance [28,29,30]. Recent studies have shown that disturbances in peroxisomal metabolism play a role in the accumulation of cellular damage due to oxidative stress and therefore, cellular aging. There is also evidence that peroxisomes can act as upstream initiators of mitochondrial ROS signaling pathways [31]. However, the precise mechanisms by which this occurs are yet to be fully elucidated.

GO analysis of downregulated datasets denoted alterations in the regulation of transcription, lipid metabolism and cell signaling (Figure 5B). Several processes related to activation and regulation of the inflammatory response/immunity were also downregulated. Previous studies have shown significant deficits in immunity in response to spaceflight, including suppression of proliferation and differentiation in hematopoietic stem cell lineages [32,33], as well as shifts in immune cell phenotypes characterized by increased numbers of bone marrow-derived T cells and decreased bone marrow-derived B cell populations [34]. Furthermore, studies using cluster of differentiation (CD) 34^+^ bone marrow progenitor cells revealed decreases in total cell number in microgravity samples, and additionally decreased erythropoiesis with concomitant increased macrophage differentiation [35].

### 2.4. Pathways Involved in Lipid Membrane Metabolism and Protein Biosynthesis Are Enriched in Multi-‘Omics Datasets from Livers of Spaceflight Mice

**Figure 6:**
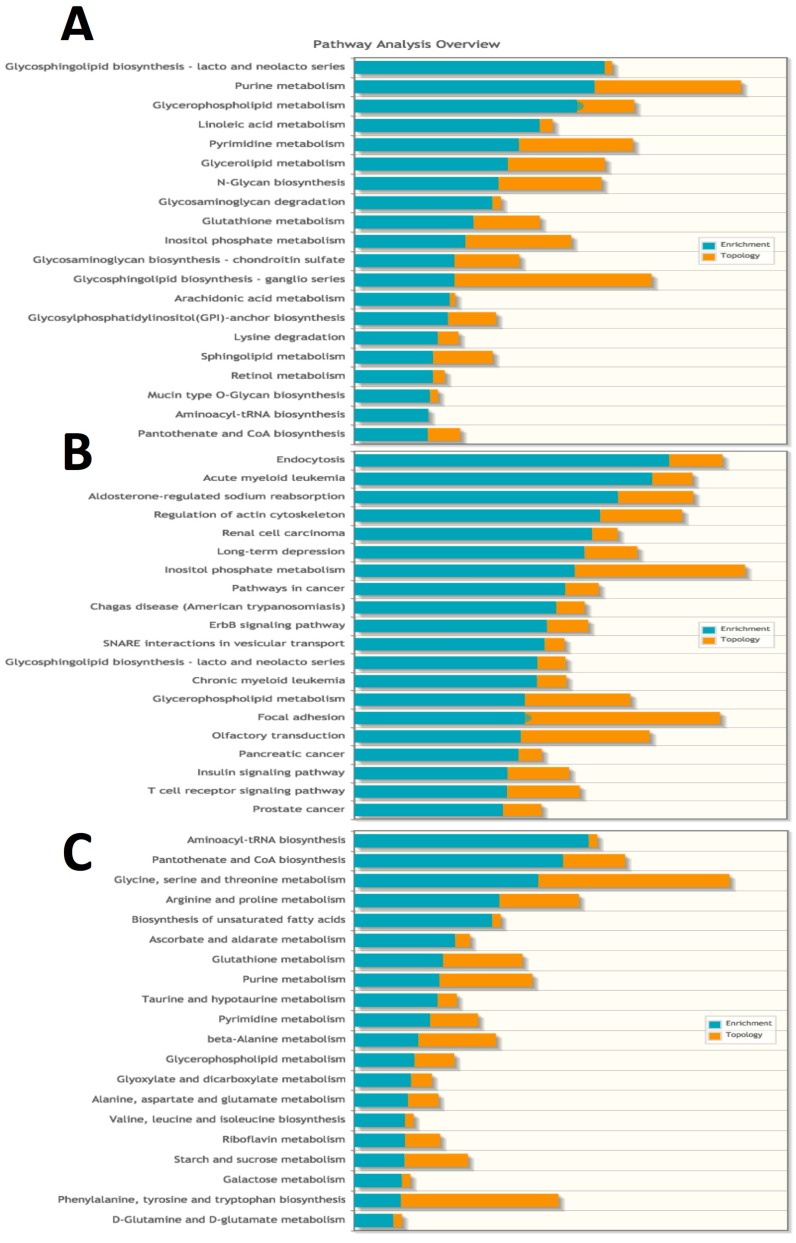
Integrated enrichment analysis using multi-‘omics datasets from livers of spaceflight mice compared with AEM ground controls. Data were submitted to the MetaboAnalyst Integrated Pathway Analysis module. (**A**) gene-metabolite; (**B**) gene and (**C**) metabolite centric workflows were compared for *n* = 6 mice per group.

To further validate our metabolomics results, we performed an integrated analysis of transcriptomics and metabolomics datasets using the MetaboAnalyst Integrated Analysis function. This algorithm performs an enrichment analysis to determine whether the observed genes and metabolites in a given pathway appear more often than expected by random chance within the dataset. An additional topology analysis evaluates whether a given gene or metabolite plays an important role in a biological response based on its position within a pathway. An over-representation analysis based on hypergeometric testing using 17,403 genes and 247 metabolites was used for the enrichment analysis and topology was assessed with “Betweenness Centrality”, which measures the number of shortest paths from all nodes to all others passing through a given node within the pathway. Integrated analysis of both genes and metabolites (Figure 6A) confirmed the impact of spaceflight on lipid membrane metabolism. Eight of the top 20 enriched pathways relate to lipid membrane metabolism (including *glycosphingolipid biosynthesis*, *glycerophospholipid metabolism*, *arachidonic acid metabolism*, *inositol phosphate metabolism*, *glycophosphatidylinositol-anchor biosynthesis* and *sphingolipid metabolism*). Previously, we reported that Ingenuity Pathway Analysis revealed *endocytosis* as an enriched gene pathway [4], which was also the most highly enriched pathway in the gene-centric analysis (Figure 6B). Enrichment of inflammatory pathways was also evident by the presence of multiple pathways related to cancer. Supporting our other metabolite set enrichment analysis, *glycine*, *serine and threonine metabolism* had high enrichment and topology scores in the metabolite-centric analytical workflow (Figure 6C).

Interestingly, several studies have shown that peroxisomes may alter lipid production and concentration in response to changes in metabolism, mediating cellular signaling through sphingolipids. In our analyses, we observed enrichment of *sphingolipid biosynthesis* and *sphingolipid metabolism*, as well as alterations in peroxisome gene expression levels, suggesting that spaceflight may alter peroxisome-related signaling pathways, including sphingolipids, to regulate cellular processes [36]. Sphingolipids, specifically, are important messengers for signaling events resulting in activation of cellular proliferation, differentiation or senescence [27,36]. Furthermore, sphingolipids have been linked to insulin resistance, oxidative stress and lipid peroxidation in hepatocytes, suggesting a potential role of sphingolipids in the progression of nonalcoholic fatty liver disease [37]. These results therefore indicate a potential connection between peroxisome redox metabolism and mitochondrial oxidative stress mediated by sphingolipid signaling pathways and resulting in upregulation of inflammatory/stress-related signaling, such as NF-κB.

Of note, *aminoacyl-tRNA biosynthesis* emerged as the top enriched metabolite pathway in the metabolite-centric analysis which, when taken together with enrichment of *purine metabolism* in the gene-metabolite centric analysis, suggested that spaceflight may increase biosynthesis or even post-transcriptional modification of tRNAs, the building blocks of mRNA decoding and protein translation. Post-transcriptional modification of tRNAs critically influences tRNA functions such as folding, stability and decoding [38,39]. Defects in tRNA modifications and modification enzymes are associated with oxidative stress [40] and human diseases including cancer, diabetes and cardiomyopathy [38]. Indeed, results from a recent study in *Caenorhabditis elegans* associating defects in post-transcriptional modification of mitochondrial tRNAs with dysfunctional oxidative phosphorylation suggest that the cell's maladaptive response to hypomodified mitochondrial tRNAs may be a mechanism underlying disease development [41]. Although speculative, the idea that exposure to the space environment may lead to aberrant tRNA post-transcriptional modifications is provocative and warrants further investigation.

**Figure 7:**
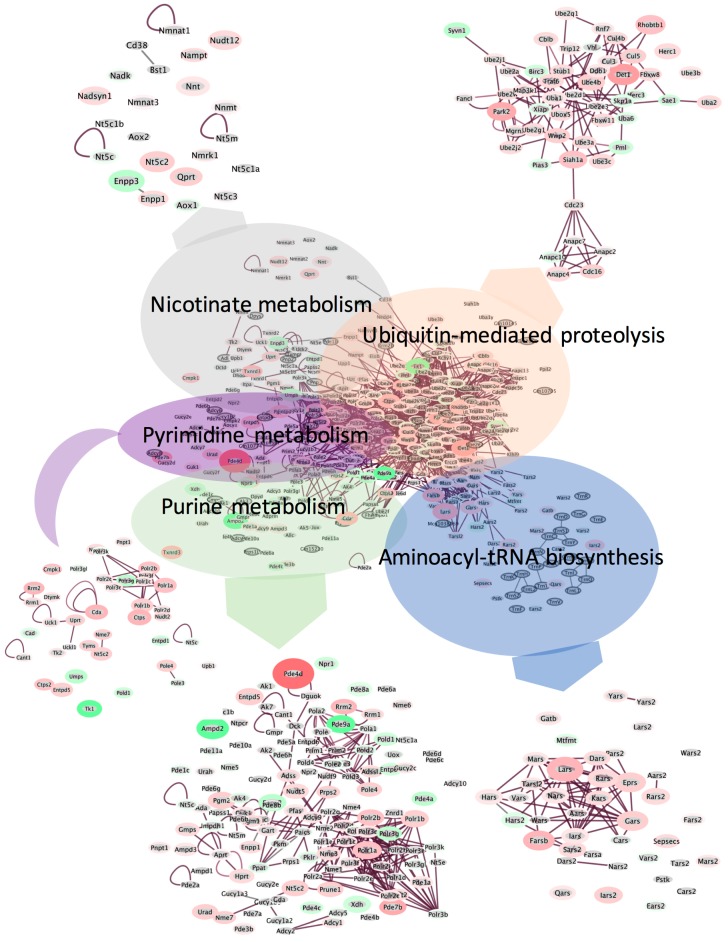
Cluster analysis using EGAN (Exploratory Gene Association Networks) software was performed using all significantly changing transcripts (*p* < 0.05). Genes associated with the top enriched pathways were clustered using a radial force-driven display. Insets are zoomed out views of each cluster. Only significantly changing genes are included in insets for clarity of presentation. Intensity of color (red = upregulated, green = downregulated, grey = not detected) is associated with degree of fold change and width of the bounding circle is inversely related to *p (corr)*-value. *n* = 6 animals per group were used for the analysis.

Since MetaboAnalyst preferentially utilizes metabolic pathways for enrichment analyses, we further interrogated the transcriptomic dataset using EGAN (Exploratory Gene Association Networks) to confirm importance of enriched pathways [42]. We performed an association analysis using all transcripts significantly changing between groups (Figure 7). Three main clusters emerged. The first cluster focused on nucleic acid metabolism and included *purine metabolism*, *pyrimidine metabolism* and *nicotinate and nicotinamide metabolism*. *Purine metabolism* enrichment was dominated by strong upregulation of phosphodiesterase 4D (*Pde4d*, Table 3), an enzyme with 3′,5′-cyclic-adenosine monophosphate (AMP) phosphodiesterase activity that degrades cAMP, an important second messenger mediating signaling in multiple pathways. Adenosine monophosphate deaminase 2 (*Ampd2*, Table 3), an enzyme that converts AMP to inosine monophosphate (IMP) and maintains cellular guanine nucleotide pools [43], was downregulated, potentially attenuating protein synthesis. This protein also mediates gluconeogenesis in the rodent liver [44] and, together with *Pde4d* upregulation (Table 3), suggests pathways impacting cellular quiescence may be altered by spaceflight.

Previous studies in spaceflight have noted alterations in gene expression related to quiescence and senescence pathways in multiple tissues. Specifically, exposure of bone marrow-derived mesenchymal stem cells to spaceflight following addition of osteogenic differentiation factors resulted in increased expression of genes related to neural development, neural morphogenesis and transmission of nerve impulses and synapses in studies conducted on the International Space Station [45]. This same study found increased expression of cell cycle arrest molecules indicating either increased differentiation of cells in space or activation of cellular quiescence or senescence [45]. Our previous studies have also found significant alterations in the proliferation and differentiation potential of both embryonic and bone marrow stem cells during 13–15 days of spaceflight, with upregulation of the cell cycle arrest and senescence marker cyclin dependent kinase inhibitor (CDKN)1a/p21 [46,47,48,49]. Notably, analysis of our liver transcriptome dataset also found upregulation of CDKN1a/p21 (3.15 fold, *p* (*corr*) < 0.05, Table 3), as well as upregulation of INK4C/p18 with concomitant attenuation of cyclin gene expression (Figure S4). Similarly, GeneSet Enrichment Analysis (GSEA) using our whole transcriptome profile showed alterations in several pathways, including quiescent cell activation, cell cycle regulation, and activation of oxidative phosphorylation. When taken in total, these results suggest potential systemic induction of cellular senescence due to short-term exposure to the space environment.

The second major cluster that emerged from our EGAN analysis was *aminoacyl-tRNA biosynthesis*, supporting the MetaboAnalyst enrichment results, showing upregulation of expression of all genes within the cluster. Modifications regulate the turnover of RNAs, and improperly modified tRNAs are targeted for degradation [50], therefore it is possible that upregulation of aminoacyl-tRNA biosynthesis actually targets cells for attenuation of protein translation due to hypomodifications or alterations of the epitranscriptome. Finally, we observed a third cluster of genes corresponding to enrichment of *ubiquitin-mediated proteolysis*. We performed Ingenuity Pathway Analysis as shown in Figure S5 and determined that the components of the 20S proteasome were significantly upregulated, supporting the observed pattern of molecular catabolism. Analysis of significantly upregulated datasets using GO analysis also indicated activation of catabolism, primarily in ATP-dependent and glycolytic pathways.

**Figure 8:**
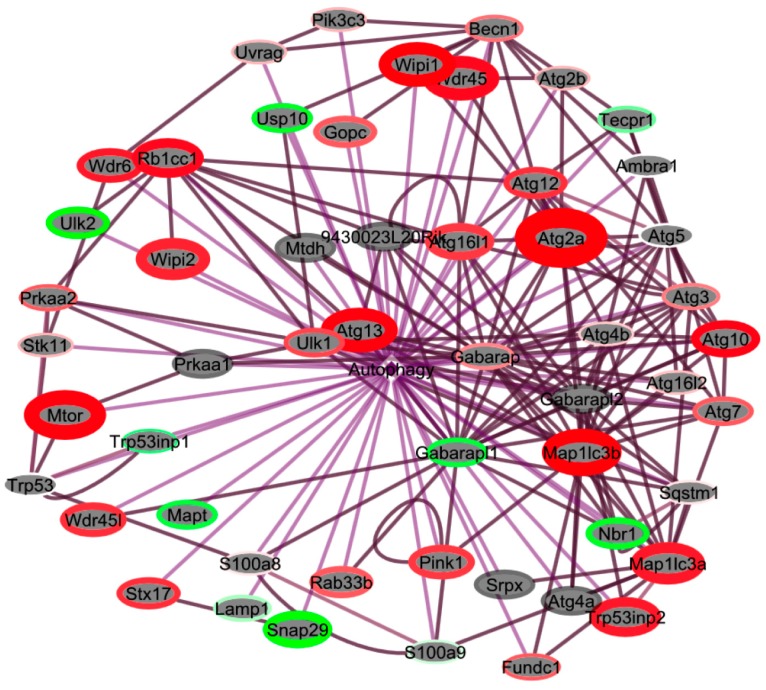
Autophagy programs are upregulated in livers from spaceflight mice. Cluster analysis using EGAN software was performed using genes associated with autophagy programs generated by BIOMART. Genes were clustered using a radial force-driven display. Intensity of color (red = upregulated, green = downregulated, grey = not detected) is associated with degree of fold change and width of the bounding circle is inversely related to *p (corr)*-value. *n* = 6 animals per group were used for the analysis.

The ubiquitin–proteasome system (UPS) and autophagy are the two main intracellular degradation pathways [51]. Autophagy primarily mediates the degradation of long-lived proteins and organelles, maintaining intracellular homeostasis. Since activation of the proteasome is associated with increased autophagy, we used BIOMART [52] to obtain a list of genes associated with autophagy GO terms and screened that list for expression changes using EGAN (Figure 8). We found significant upregulation in expression of a number of genes, including *Atg2a*, important for autophagosome formation as well as regulation of lipid droplet morphology and dispersion; microtubule-associated genes (*Map1lc3a*, *Map1lc3b*); *Wipi1* and *2*, involved in pre-autophagosome formation and the autophagy response to starvation; and *Mtor*, a central mediator of cellular response to stressors such as DNA damage and oxidative stress, supporting our GSEA results (Table 3). Alterations in the rate of autophagy have been shown to regulate ROS formation and redox balance under specific circumstances [53]. However, proteins damaged by ROS/RNS form protein aggregates that are degraded through the 20S proteasome in order to maintain cellular homeostasis [53]. Exposure to chronic or sustained oxidative stress can lead to inactivation of the proteasome, resulting in accumulation of protein conjugates. Heavily oxidized protein aggregates are also not suitable for degradation by the proteasome [54]. These aggregates may be specific targets of autophagy-related pathways mediated by heat shock protein chaperones, which were also altered in our analyses (Table 3). These results suggest that spaceflight leads to upregulation of multiple autophagy-related pathways and are consistent with activation of the proteasome and attenuation of protein synthesis in livers from FLT mice as compared with AEM controls.

**Figure 9:**
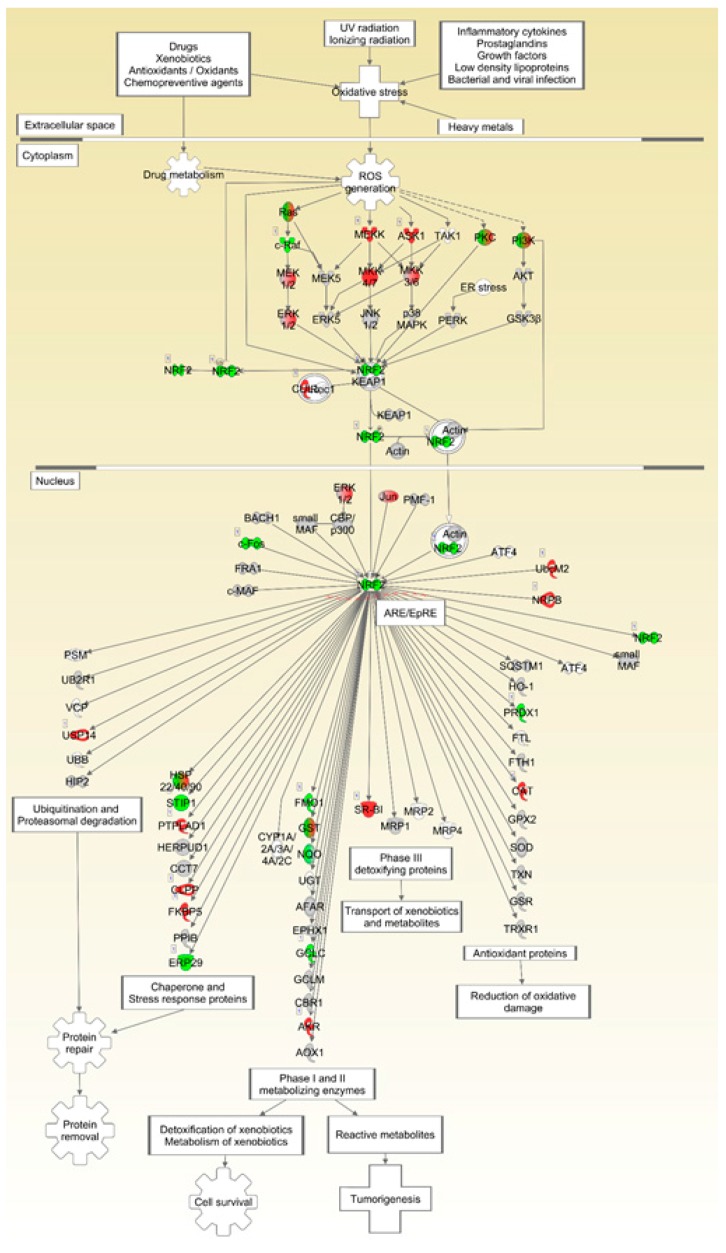
NFE2L2/NRF2-mediated pathways are downregulated in spaceflight mouse livers. Ingenuity Pathway Analysis was used for analysis of mRNA transcript levels in livers from FLT and AEM control mice. Grey = unchanged, green = downregulated, red = upregulated. Intensity of color correlates with degree of fold-change. *n* = 6/group.

Finally, we sought to determine whether decreased response to oxidative stress was associated with induction of autophagy and we performed a pathway analysis of the nuclear factor (erythroid-derived 2)-like 2 (NFE2L2/NRF2)-mediated response to oxidative stress (Figure 9). We found significant downregulation of *Nrf2* expression levels in FLT mice as compared to AEM controls (Table 3), as well as diminished expression of downstream pathway members, suggesting that exposure to the space environment leads to attenuation of oxidative defense. Notably, spaceflight studies using mice flown on STS-131 for 15 days also revealed downregulation of this oxidative stress mitigator in bone marrow tissues [46,47]. Unfortunately, we did not have sufficient sample to directly probe for changes in protein oxidation, although oxidative damage is a likely cause for the observed increase in autophagy programs and upregulation of the proteasome in the FLT mice.

Autophagy-related programs are essential for liver regeneration [55] and repair and induction of autophagy may occur in response to exposure to the space environment. Several studies have characterized the molecular mechanisms involved in regeneration of the liver in response to a variety of stress conditions and in response to partial hepatectomy (PHx), whereby a significant portion of the rodent liver is removed and the remaining portion regenerates and restores the liver to its original size. This process is highly regulated and includes several distinct stages, including withdrawal of hepatocytes from quiescence, cell cycle entry and progression, cessation of cell division and return of hepatocytes to quiescence [56,57,58,59]. Although hepatocytes are the first cells to replicate, they are followed sequentially by other cell types within the liver including stellate cells and sinusoidal endothelial cells [59].

A recent study demonstrated that autophagy is critical in the prevention of hepatocyte senescence during the early stages of liver regeneration, and inhibition of autophagy-related genes results in delayed liver regeneration, aggregation of unfolded proteins, and activation of senescence in hepatocytes with a corresponding increase in senescence-associated secretory phenotype (SASP)-related molecules [58]. This also coincides with considerable damage to the mitochondria, reduced β-oxidation and reduced intrahepatic ATP generation, leading to dysregulation of hepatocellular lipid stores [58]. As we found mild upregulation of autophagy-related genes as well as upregulation of senescence, it is possible that autophagy pathways were initially activated to degrade oxidized proteins. However, as chronic oxidative stress has been shown to induce hepatocyte senescence, it is likely that senescence signaling pathways were activated in response to accumulation of oxidized proteins and failure of autophagy mechanisms to clear these proteins. Indeed, other studies have also demonstrated links between increased oxidative stress and increased hepatocyte senescence, resulting in steatosis due to mitochondrial dysfunction [60,61,62]. This senescence-associated mitochondrial dysfunction is a regulated process driven by signaling through p21 and through p38 mitogen-activated protein kinase (MAPK), both of which we found to be upregulated in the current study (Table 3). Such changes are very similar to those that occur during ageing and obesity-related pathologies, such as insulin resistance, and have been associated with impaired energy generation and increased production of ROS. Studies conducted by us and others have shown spaceflight leads to increased oxidative stress, increased insulin resistance, and systemic induction of aging-related pathologies. It is possible that in the liver, increased oxidative stress and altered autophagy pathways may cause hepatocyte senescence through activation of p21 and mitochondrial dysfunction, resulting in hepatic steatosis (as we previously reported [5]) and impaired regenerative capacity. These molecular changes may have important implications for the onset of obesity-related diseases and regenerative health in the course of long-duration space exploration.

## 3. Materials and Methods

Animal studies were reviewed and approved by multiple Animal Care and Use Committee (ACUC) boards, including the NASA Ames Research Center ACUC (NAS-11-002-Y1; 31 May 2011), the NASA Kennedy Space Center (KSC) ACUC (FLT-11-078; 23 May 2011) and the University of Colorado at Boulder Institutional ACUC (1104.11; 10 May 2011). No protocol was required for assays performed at the University of Colorado Anschutz Medical Center or Loma Linda University since only tissues obtained after euthanasia (no live animals) were analyzed at our sites. All NASA studies involving vertebrate animals were carried out in strict accordance with the recommendations in the Guide for the Care and Use of Laboratory Animals of the National Institutes of Health.

### 3.1. Animals and Sample Collection

Nine-week old weight-matched female C57BL/6J mice were selected for this study because they produce fewer odor annoyance issues and were the only gender approved for flight (*n* = 15/group). Additionally, all historical data (e.g., STS-118) were obtained on female mice so the use of female mice permitted comparisons across missions, particularly for the musculoskeletal studies that were primary. Mice were housed in Animal Enclosure Modules (AEMs, 10 mice per AEM habitat; 5 per side separated by a wire mesh) and either flown on the Space Shuttle Atlantis (STS-135) for 13.5 days (FLT) or housed at the Space Life Science Laboratory (SLSL) at KSC (Ground AEM controls; AEM).

Following two days of acclimation after receipt, mice were provided a NASA NuRFB foodbar (TD 04197; 47% carbohydrate, 17.9% protein, 3.9% lipids, 2.8% fiber, 2.80 kcal/g). All mice were placed into AEM housing one day before flight. Environmental parameters for ground control mice were matched as closely as possible with flight conditions using 48 h delayed telemetry data. Conditions were controlled for temperature, humidity, and a 12/12 h light/dark cycle; foodbars and water were provided *ad libitum*.

Tissues were harvested at the SLSL within 3–5 h after return of the Space Shuttle Atlantis and were distributed amongst a team of investigations through NASA’s Biospecimen Sharing Program; we received one half lobe of liver from six mice per group. Mice were euthanized using 4% isoflurane followed by cardiac puncture and exsanguination. Liver lobes were extracted and dissected. A portion of the liver was prepared in 4% paraformaldehyde and the rest snap frozen in liquid nitrogen then shipped to either Loma Linda University or University of Colorado Anschutz Medical Campus and stored appropriately prior to use.

### 3.2. Transcriptomics

Samples were prepared and analyzed as previously described [4,5]. Briefly, RNA was isolated using an RNeasy kit (Qiagen, Germantown, MD, USA) and the Ambion WT expression kit (Thermo Fisher Scientific, Waltham, MA, USA) was employed to prepare mRNA for whole transcriptome microarray analysis using an Affymetrix GeneChip 1.0 ST array (Thermo Fisher Scientific). Arrays were scanned using a GeneChip Scanner 3000 7G (Thermo Fisher Scientific) and Command Console Software v. 3.2.3 (Thermo Fisher Scientific) to produce. CEL intensity files which were processed with CARMAweb (Comprehensive R-based Microarray Analysis web service). GeneSpring software (Agilent, Santa Clara, CA, USA) was used to perform statistical analysis. Specifically, samples were first filtered on signal intensity values in order to remove background noise, statistical analysis was performed on normalized and filtered samples using a moderated *t*-test with Benjamini Hochberg FDR correction factor. Samples were then filtered based on fold change, whereby *p* (corr) < 0.05 and fold change +/− 1.5 or more was considered significant. CARMAweb files were then imported into Ingenuity Pathway Analysis (Qiagen) software for subsequent analysis.

### 3.3. Metabolomics

Frozen liver pieces (*n* = 6/group) were shipped to Metabolon, Inc. (Morrisville, NC, USA) and stored at −80 °C before use. Samples were prepared for the appropriate instrument, either Liquid Chromatography/Mass Spectrometry (LC/MS) or Gas Chromatography/Mass Spectrometry (GC/MS), as described previously [4,5]. Briefly, automated sample preparation was conducted to extract metabolites for analysis by LC and GC. Extracts were placed briefly on a TurboVap^®^ (Zymark, Clackamas, OR, USA) to remove organic solvent. Each extract was then frozen and dried under vacuum.

### 3.4. Liquid Chromatography/Mass Spectrometry (LC/MS)

Extracts were split into two aliquots, dried, then reconstituted in acidic or basic LC-compatible solvents, each of which contained 11 or more injection standards at fixed concentrations. Metabolite features were measured using a Waters ACQUITY UPLC (Waters, Milford, MA, USA) and a Thermo-Finnigan LTQ-FT MS (Thermo Fisher Scientific) in two independent injections using separate dedicated columns as described previously [4,5]. One aliquot was analyzed in positive ion mode under acidic conditions and gradient eluted from a Waters BEH C_18_ 2.1 mm × 100 mm column, containing 1.7 µm resin, using water and methanol as mobile phases with both containing 0.1% formic acid. The other aliquot was analyzed in negative ion mode using basic conditions, which also employed water/methanol, and contained 6.5 mM ammonium bicarbonate for ion pairing. Mass spectrometric analysis alternated between MS and data-dependent MS^2^ scans using dynamic exclusion, scanning from 80–1000 *m*/*z*. Accurate mass measurements were made on precursor ions with greater than 2 million counts; typical mass error was less than 5 ppm.

### 3.5. Gas Chromatography/Mass Spectrometry (GC/MS)

Extracts destined for GC/MS were vacuum desiccated for 24 h then derivatized under dried nitrogen using bis(trimethylsilyl)-trifluoroacetamide (BSTFA). Volatile metabolites were separated using 5% phenyl/95% dimethyl polysiloxane fused silica (20 m × 0.18 mm ID; 0.18 µm film thickness) and a temperature ramp from 40 to 300 °C within 16 min; helium was used as the carrier gas. Compounds were analyzed using a Thermo-Finnigan Trace DSQ fast-scanning single-quadrupole MS (Thermo Fisher Scientific) equipped with electron impact ionization set to scan from 50–750 *m*/*z* at unit mass resolving power, as we previously described [4,5].

### 3.6. Compound Identification

Mass spectral data were loaded into a relational database and peaks were identified using Metabolon’s peak integration software [63]. Compounds were identified by comparison to library entries of purified standards based on the combination of retention time and mass spectra. Data were normalized to correct for variation resulting from instrument inter-day tuning differences. Each compound was corrected by registering the medians to equal 1.00 in run-day blocks and normalizing each data point proportionately. Missing values, assumed to be below the limit of detection of the instrument, were imputed with the observed minimum after normalization.

### 3.7. Data Availability

Transcriptomics and metabolomics data are publicly accessible via the NASA GeneLAB data base. Transcriptomics data are found at the website (Available online: https://genelab-data.ndc.nasa.gov/genelab/accession/GLDS-25/). Metabolomics data are available at the website (Available online: https://genelab-data.ndc.nasa.gov/genelab/accession/GLDS-108/).

### 3.8. Integrated Data Analysis

MetaboAnalyst software (Available online: www.metaboanalyst.ca) was used for pathway and enrichment analysis of metabolomics data as well as integrated analysis of transcriptomics and metabolomics datasets. Data were log-transformed and auto-scaled then subjected to various analytical modules within the software [64]. Ingenuity Pathway Analysis (IPA) and Exploratory Gene Association Networks (EGAN) [42] (Available online: akt.ucsf.edu/EGAN) were used to assess pathway activation based on changes in mRNA expression levels. Human Metabolite Database (HMDB) accession numbers were queried and names of genes associated with metabolites exhibiting significant changes in abundance were extracted manually. These genes were used to construct a subset list of genes that may be functionally significant, based on metabolite abundance changes, and this subset was also subjected to pathway and enrichment analysis.

### 3.9. Histology

Fixed tissue sections were processed for hematoxylin and eosin (H&E) staining as described previously [65]. Histologic images were captured on an Olympus BX51 microscope equipped with a 4mp Macrofire digital camera (Optronics, Tokyo, Japan) using the PictureFrame Application 2.3 (Optronics). All images were cropped and assembled using Photoshop CS2 (Adobe Systems, Inc., San Jose, CA, USA).

### 3.10. Statistical Analysis

Data were analyzed with GraphPad Prism V6.0 (GraphPad Software, La Jolla, CA, USA) using a Mann-Whitney *U*-test or unpaired *t*-test with Welch’s correction for comparison between groups. Means ± SEM were reported. The ROUT method with *Q* = 1% was used to identify outliers for exclusion from analysis. *p (corr)*-values less than 0.05 were selected to indicate significance.

## 4. Conclusions

The unbiased analyses presented here both support our previous results and extend them to show that exposure to the space environment for only 13.5 days results in increased oxidative stress due to elevated ROS and impaired oxidative defense (by way of attenuation of NRF2-related pathways) in the mouse liver. Furthermore, our multi-‘omics studies suggest that accumulation of oxidized proteins coupled with mitochondrial dysfunction may lead to activation of hepatocyte senescence, resulting in hepatocyte lipid accumulation and steatosis. Further investigation into the potential for liver damage in the course of long-duration space exploration is needed, as are development of effective countermeasures to protect astronaut health.

## Figures and Tables

**Table 1:** Significantly changing biochemicals in spaceflight identified in volcano plot analysis.

Biochemicals	FC	log2 (FC)	*p*	−log10 (*p*)
Reduced				
4-Guanidinobutanoate ^1,^*	0.377	−1.409	1.98 × 10^−5^	4.703
Glycerophosphorylcholine *	0.282	−1.826	1.52 × 10^−3^	2.817
3-Ureidopropionate	0.466	−1.102	3.83 × 10^−2^	1.417
Increased				
3-hydroxybutyrate *	3.229	1.691	7.54 × 10^−5^	4.123
Glutarate pentanedioate *	4.766	2.253	8.40 × 10^−5^	4.076
Propionylcarnitine *	2.440	1.287	1.68 × 10^−4^	3.776
3-methylglutarylcarnitine *	3.478	1.798	5.90 × 10^−4^	3.229
Dimethylglycine *	2.195	1.134	1.87 × 10^−3^	2.728
Hexadecanedioate *	2.189	1.131	2.07 × 10^−3^	2.684
Ophthalmate	2.326	1.218	1.30 × 10^−2^	1.887
Hydroxyisovaleroyl carnitine	2.567	1.360	1.37 × 10^−2^	1.862
Putrescine	2.857	1.514	3.48 × 10^−2^	1.459
Cholate	2.602	1.380	4.98 × 10^−2^	1.302
Taurodeoxycholate	2.978	1.574	5.00 × 10^−2^	1.301

^1^, biochemical with the most significant change in volcano plot and FC analysis. *, indicates biochemical is also one of the top 15 VIP features contributing to the PLS-DA analysis. FC–fold change comparing FLT to AEM controls. *n* = 6/group.

**Table 2:** Average food and water intake measurements for STS-135 mice and AEM controls.

Intake	AEM ^a^	FLT	FLT/AEM	*p*-Value
Food Intake (g) ^b^	4.08 ± 0.10	4.09 ± 0.18	1.00	0.865
Water Intake (g)	3.38 ± 0.22	2.73 ± 0.01	0.81	0.038

^a^, Food and water consumption were measured over the 13.5-day flight. Values represent mean ± SEM; ^b^, Intake values are means calculated for 3 cages of 5 mice per group. Table adapted from [5].

**Table 3:** Differentially regulated genes and biological processes in livers of FLT mice.

Gene Name	Gene ID	*p (corr*) Value	Fold Change	GO Biological Process
*Agpat9*	1-Acylglycerol-3-phosphate-*o*-acyltransferase 9	8.10 × 10^−4^	3.740	Lipid metabolic process
*Cdkn1a*	Cyclin-dependent kinase inhibitor1A (P21)	4.24 × 10^−4^	3.153	Regulation of cyclin-dependent protein serine
*Elovl3*	Elongation of very long chain fatty acids-like3	3.76 × 10^−3^	3.055	Lipid metabolic process
*Pnpla2*	Patatin-like phospholipase domain containing 2	1.17 × 10^−3^	2.645	Lipid metabolic process
*Pde4d*	Phosphodiesterase 4D, cAMP specific	7.68 × 10^−4^	2.161	cAMP catabolic process
*Pex11a*	Peroxisomal biogenesis factor 11 alpha	1.34 × 10^−3^	2.066	Peroxisome organization
*Pex3*	Peroxisomal biogenesis factor 3	8.82 × 10^−3^	2.037	Peroxisome organization
*Pex19*	Peroxisomal biogenesis factor 19	3.23 × 10^−3^	1.953	Protein targeting to peroxisome
*Cirbp*	Cold inducible RNA binding protein	1.15 × 10^−2^	1.895	Response to stress
*Pex16*	Peroxisomal biogenesis factor 16	4.73 × 10^−4^	1.887	Protein targeting to peroxisome
*Acot8*	Acyl-Coa-thioesterase 8	4.73 × 10^−4^	1.776	Peroxisome organization
*Atg2a*	Autophagy related 2A	2.59 × 10^−3^	1.707	Autophagy
*Vwa8*	Von willebrand factor A domain containing 8	1.94 × 10^−3^	1.651	ATP catabolic process
*Abcd3*	ATP-binding cassette, sub-familyD (ALD), member 3	1.94 × 10^−3^	1.651	ATP catabolic process
*Abcg8*	ATP-binding cassette, sub-family G (WHITE), member 8	2.08 × 10^−2^	1.628	ATP catabolic process
*Atp10d*	ATPase, class V, type 10D	7.48 × 10^−3^	1.614	ATP catabolic process
*Map1lc3b*	Microtubule-associated protein 1 light chain 3 β	2.50 × 10^−2^	1.553	Autophagy
*Abcg5*	ATP binding cassette subfamily G member 5	4.11 × 10^−2^	1.550	ATP catabolic process
*Ppara*	peroxisome proliferator activated receptor α	2.10 × 10^−3^	1.550	Negative regulation of transcription
*Mtor*	Mechanistic target of rapamycin	5.06 × 10^−3^	1.541	Positive regulation of protein phosphorylation
*Wipi1*	WD repeat domain, phosphoinositide interacting 1	3.81 × 10^−2^	1.537	Autophagic vacuole assembly
*Ppargc1b*	PPARG coactivator 1 β	1.24 × 10^−2^	1.535	Transcription from mitochondrial promoter
*Atg14*	Autophagy related 14	2.54 × 10^−2^	1.505	Autophagic vacuole assembly
*Pex1*	Peroxisomal biogenesis factor 1	8.32 × 10^−3^	1.417	Protein targeting to peroxisome
*Pex11b*	Peroxisomal biogenesis factor	2.49 × 10^−2^	1.365	Peroxisome organization
*Pex10*	Peroxisomal biogenesis factor 10	3.85 × 10^−2^	1.357	Peroxisome organization
*Wipi2*	WD repeat domain, phosphoinositide interacting 2	1.67 × 10^−2^	1.277	Autophagic vacuole assembly
*Map1lc3a*	Microtubule associated protein 1 light chain 3 α	1.30 × 10^−2^	1.256	Autophagic vacuole assembly
*Ampd2*	Adenosine monophosphate deaminase 2	1.01 × 10^−2^	−1.608	AMP biosynthetic process
*Nfe2l2*	Nuclear factor, erythroid 2 like 2	5.80 × 10^−3^	−1.643	Transcription, DNA-dependent
*Cyp26a1*	Cytochrome P450 family 26 subfamily A member 1	4.97 × 10^−2^	−2.015	Central nervous system development
*Hsp90aa1*	Heat shock protein 90 α family class A member 1	7.68 × 10^−4^	−2.653	ATP catabolic process
*Hspb1*	Heat shock protein family B (small) member 1	1.03 × 10^−3^	−5.755	Response to stress

Red indicates significantly upregulated genes in FLT compared to AEM controls, whilst blue indicates significantly downregulated genes in FLT compared to AEM controls.

## Supplementary Materials

Supplementary materials can be found at www.mdpi.com/1422-0067/18/10/2062/s1.
