# Supplementary material for: Spaceflight Activates Autophagy Programs and the Proteasome in Mouse Liver
Source: Int J Mol Sci. 2017 Sep 27;18(10):2062. doi: 10.3390/ijms18102062 (PMC5666744; doi:10.3390/ijms18102062)
Supplement: Supplementary file 1 [file ijms-18-02062-s001.pdf]

Spaceflight Activates Autophagy Programs and the Proteasome in Mouse Liver

Elizabeth A. Blaber, Michael J. Pecaut, and Karen R. Jonscher

Supplemental Information

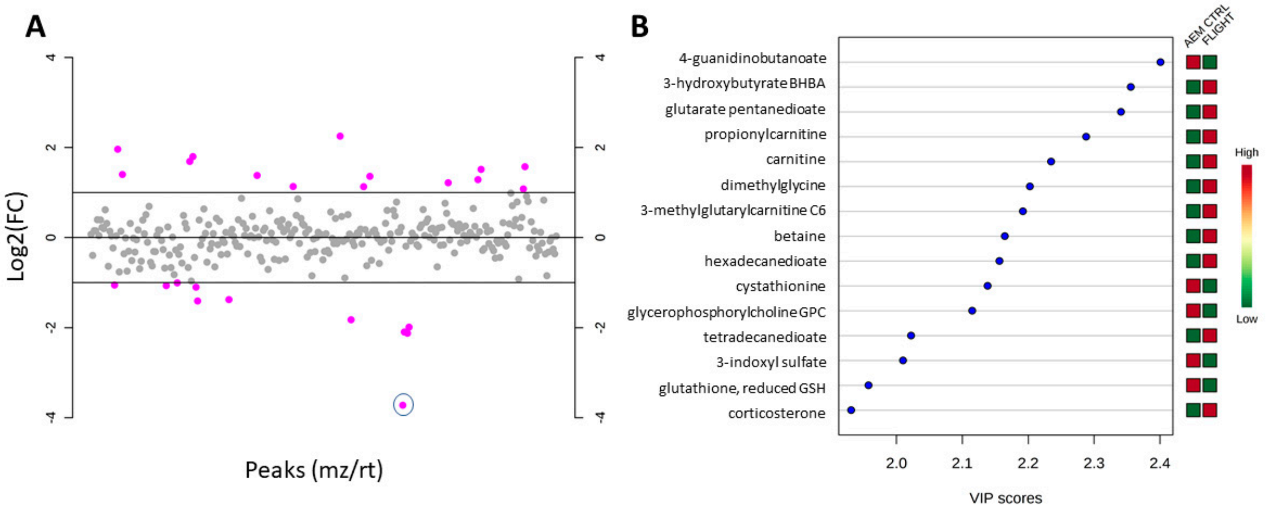

Figure S1. 4-guanidinobutanoate is the most significant contributor to differences between FLT and AEM metabolite abundances in mouse liver. A) Fold-change (FC) analysis of metabolites comparing FLT vs AEM controls. 4-guanidinobutanoate is the most diminished in abundance in FLT mice and is circled. FC=2 was selected as a cutoff for significance. B) VIP analysis showing the top 15 scoring metabolites contributing to the PLS-DA model and their relative abundance. Two thirds of the metabolites are increased in abundance in FLT mice and one third are decreased.

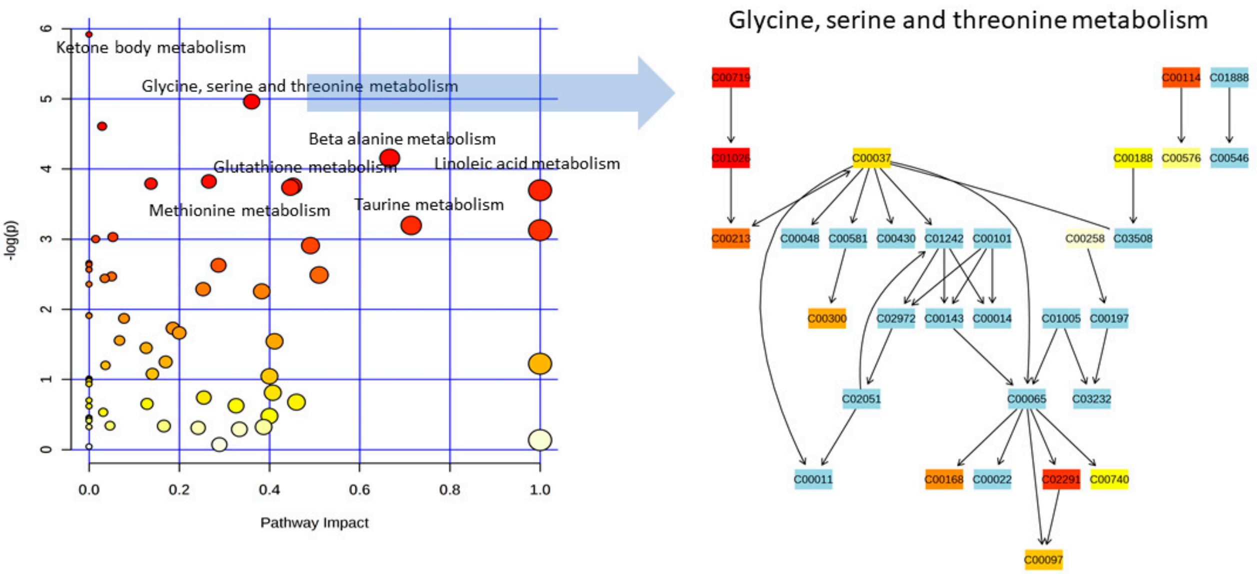

Figure S2. Metabolite-focused pathway analysis. Pathway enrichment and topology analysis was performed using MetaboAnalyst. *P*-values were obtained from the enrichment analysis and pathway impact values were calculated from the topology analysis, which used global ANCOVA and relative betweenness centrality to determine relationships between nodes. A total of 82 pathways in the *mus musculus* library were interrogated. Abundance of detected metabolites in the *glycine, serine and threonine* metabolic pathway are color coded: red – upregulated significantly, orange – not changing, yellow – downregulated, blue – not measured.

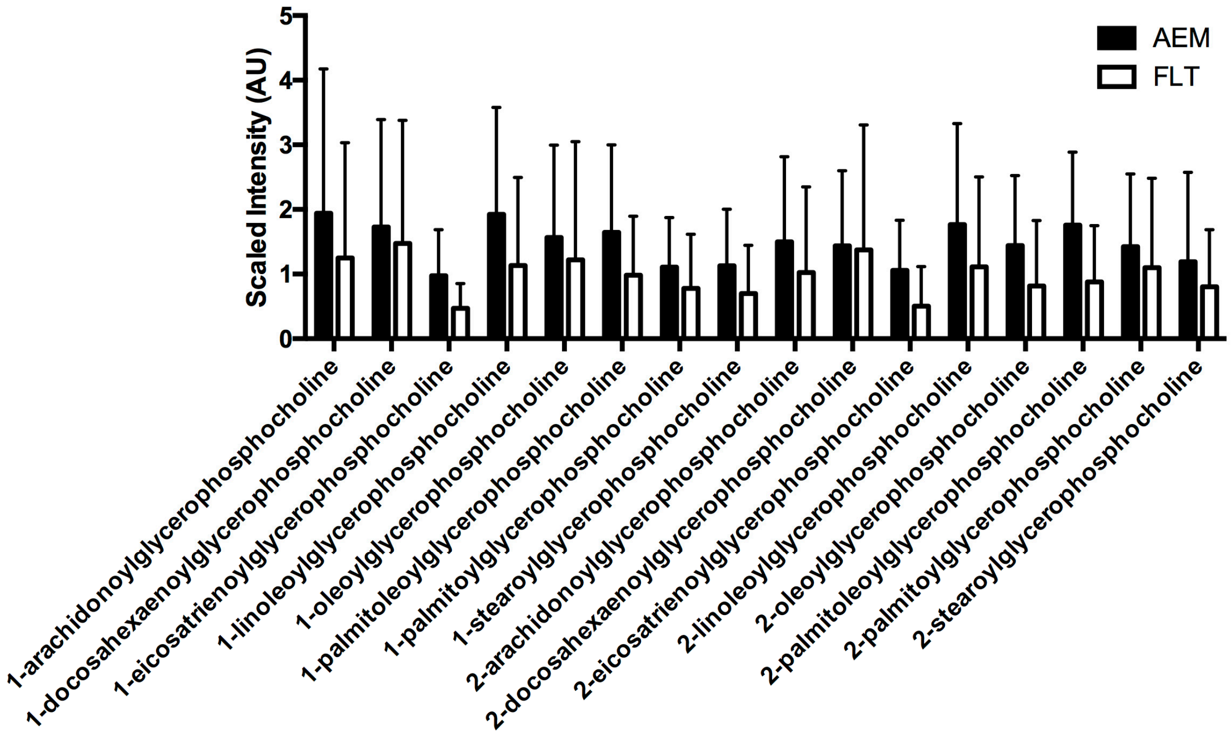

Figure S3. Abundance of choline-containing lysolipids is decreased in livers of spaceflight mice. Metabolomics analysis was performed as described in Methods and Materials on livers from *n*=6 mice per group. A two-tailed student's *t*-test was used to compare between groups. No *P*-values were obtained below 0.05.

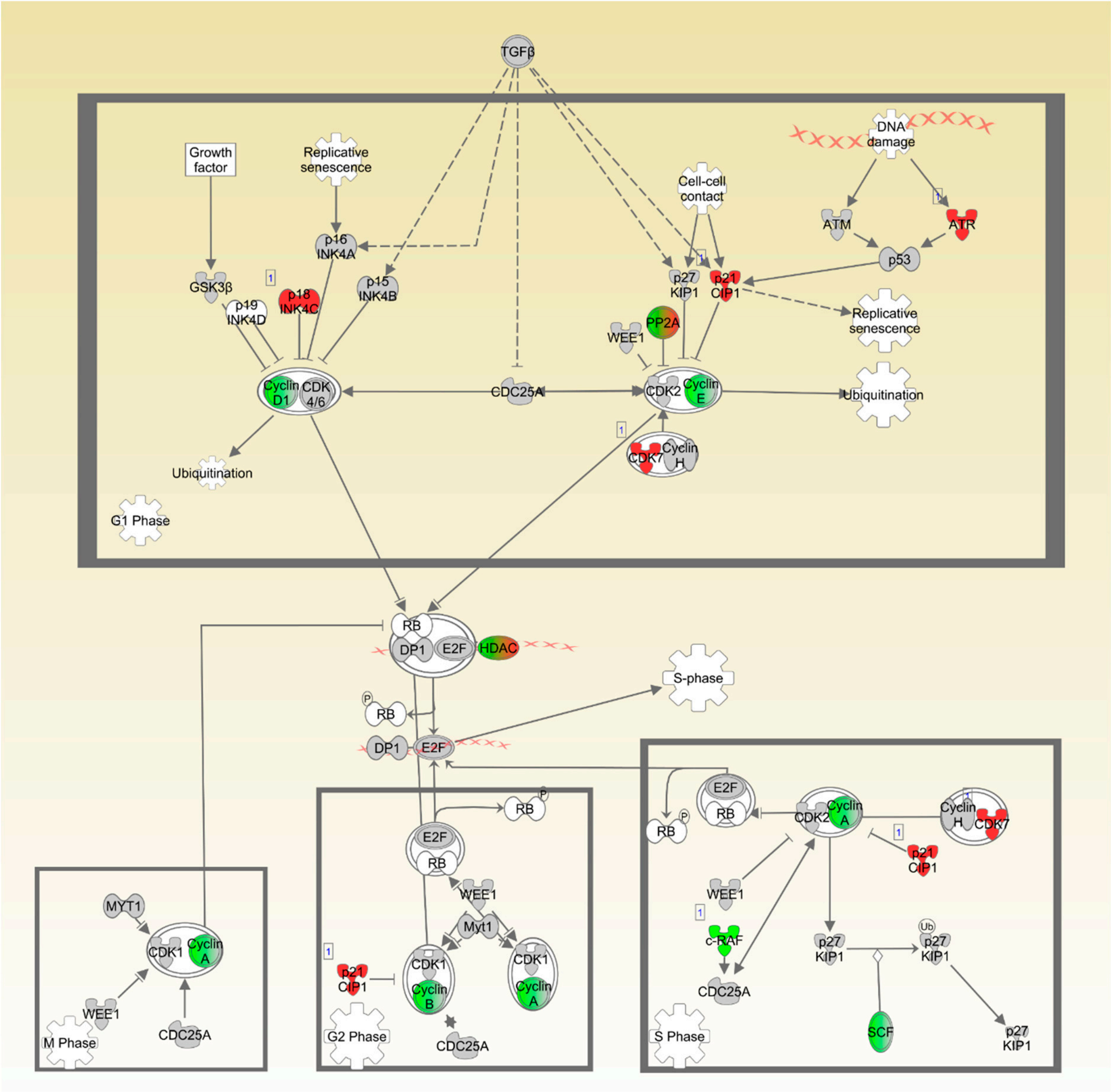

© 2000-2017 QIAGEN. All rights reserved.

Figure S4. Cell cycle is attenuated in livers of FLT mice. Ingenuity Pathway Analysis was performed using transcriptomics datasets comparing mRNA expression levels in FLT and AEM control livers. Grey = unchanged. Green = downregulated. Red = upregulated, *n*=6/group.

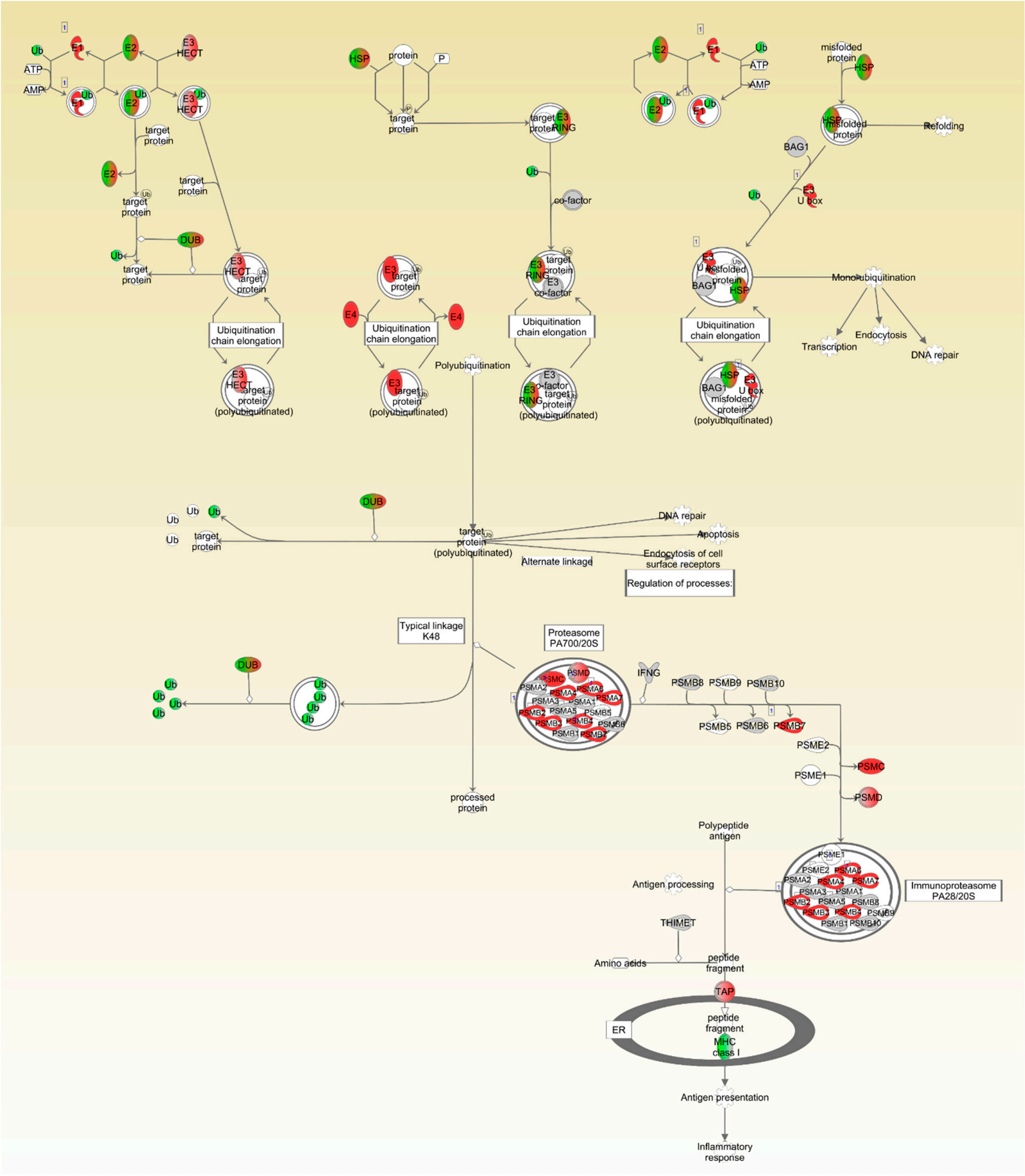

© 2000-2017 QIAGEN. All rights reserved.

Figure S5. Components of the proteasome network are upregulated in livers of FLT mice. Ingenuity Pathway Analysis was performed using transcriptomics datasets comparing mRNA expression levels in FLT and AEM control livers. Grey = unchanged. Green = downregulated. Red = upregulated, *n*=6/group.

Table S1. Fold change of biochemicals from FLT livers compared to AEM controls

	Fold Change	log2(FC)
**Reduced**		
maltohexaose	0.0757	-3.724
maltotetraose	0.2292	-2.126
maltopentaose	0.2336	-2.098
maltotriose	0.2517	-1.990
glycerophosphorylcholine GPC*	0.2820	-1.826
4-guanidinobutanoate*	0.3766	-1.409
arabonate	0.3848	-1.378
3-ureidopropionate*	0.4660	-1.102
2-eicosatrienoylglycerophosphocholine	0.4772	-1.067
1-eicosatrienoylglycerophosphocholine	0.4813	-1.055
2-palmitoleoylglycerophosphocholine	0.4978	-1.006
**Increased**		
Taurocholate*	2.1150	1.081
Hexadecanedioate*	2.1894	1.131
Dimethylglycine*	2.1950	1.134
Ophthalmate*	2.3256	1.218
Propionylcarnitine*	2.4402	1.287
hydroxyisovaleroyl carnitine*	2.5670	1.360
Cholate*	2.6025	1.380
1-oleoylglycerol 1-monoolein	2.6415	1.401
Putrescine*	2.8570	1.514
Taurodeoxycholate*	2.9778	1.574
3-hydroxybutyrate BHBA*	3.2294	1.691
3-methylglutarylcarnitine C6*	3.4779	1.798
1-linoleoylglycerol 1-monolinolein	3.8965	1.962
glutarate pentanedioate*	4.7664	2.253

* - indicates P < 0.05

Table S2. Abundance of metabolites contributing to significantly enriched metabolite sets

			Fold Change[a]			*P*-value[b]
**Biochemical Name**	**KEGG**	**HMDB**	**FLIGHT vs. AEM**	**Mean Values AEM**	**Mean Values FLIGHT**	**FLIGHT vs. AEM**
propionylcarnitine	C03017	HMDB00824	1.287	0.952	2.324	**0.0002**
dimethylglycine	C01026	HMDB00092	1.131	0.730	1.603	**0.0019**
betaine	C00719	HMDB00043	0.872	0.938	1.717	**0.0009**
carnitine	C00318		0.475	0.923	1.281	**0.0004**
5-methyltetrahydrofolate (5MeTHF)	C00440	HMDB01396	0.390	0.844	1.106	0.2210

S-adenosylhomocysteine (SAH)	C00021	HMDB00939	-0.218	1.060	0.908	0.1383
choline	C00114		-0.434	1.275	0.941	**0.0415**
cystathionine	C02291	HMDB00099	-0.474	1.176	0.841	**0.0014**
cysteinylglycine	C01419	HMDB00078	-0.515	0.981	0.690	**0.0165**
glutathione, reduced (GSH)	C00051	HMDB00125	-0.889	0.978	0.525	**0.0049**

[a] - $\log_2$ fold change values are used.; [b] - P-values calculated using Student's *t*-test.
